# Editorial Expression of Concern: Swim training improves leptin receptor deficiency-induced obesity and lipid disorder by activating uncoupling proteins

**DOI:** 10.1038/s12276-025-01589-9

**Published:** 2025-11-21

Editorial Expression of Concern to: *Experimental & Molecular Medicine* 10.1038/emm.2007.43, published online 01 June 2007

The Editor-in-Chief is issuing an Editorial Expression of Concern to alert readers that, after the publication of this article, it was brought to the attention of the publisher that, in Figure 2, the β-actin control appears to be highly similar for both male and female mice. The Female UCP3 Swim blot appears to overlap with the blot representing Female UCP2 Control. The authors were unable to provide the original images due to the passage of time. Readers are urged to take caution when interpreting the content and conclusions of this article.

The original article can be found online at 10.1038/emm.2007.43.

